# The Impact of Age Differences and Injury Severity on Pedestrian Traffic Crashes: An Analysis of Clinical Characteristics and Outcomes

**DOI:** 10.3390/jcm14030741

**Published:** 2025-01-23

**Authors:** Rayan Jafnan Alharbi

**Affiliations:** Emergency Medical Services Program, Department of Nursing, College of Nursing and Health Sciences, Jazan University, Jazan 45142, Saudi Arabia; rjalharbi@jazanu.edu.sa

**Keywords:** pedestrian traffic crashes, injury severity, age differences, clinical outcomes, injury epidemiology, demographic trends, injury patterns, pedestrian safety strategies

## Abstract

**Background/Objectives**: The incidence of pedestrian traffic injuries is an escalating concern for public health worldwide. Particularly in fast-developing nations, such as Saudi Arabia, these injuries form a significant portion of trauma-related healthcare challenges. This study aims to explore age-specific differences in trends, seasonal variations, and the overall impact of pedestrian traffic injuries in Riyadh, Saudi Arabia, with a focus on injury characteristics and clinical outcomes. **Methods**: The study conducted a retrospective analysis using data from the Saudi Trauma Registry (STAR) covering the period between August 2017 and December 2022. It employed descriptive statistics, chi-square tests, and multivariable linear regression analyses to explore demographic trends, characteristics of injuries, and hospital-based outcomes. **Results**: This study analyzed data from 1062 pedestrian injury cases, revealing key demographic and clinical patterns. Most incidents occurred on weekdays (71.9%) and during nighttime hours (63.3%), with seasonal peaks observed from April to June (30.4%). The lower extremities (27.5%) and head (21.3%) were the most frequently injured body regions. ICU admissions were more common among individuals aged 30–40, females, and those with head or chest trauma, while higher in-hospital mortality was associated with patients over 60 years old, transport by private or police vehicles, and extended ICU and hospital stays. Approximately 25.6% of cases required ICU care, with an overall in-hospital mortality rate of 4.9%. **Conclusions**: This study provides an in-depth analysis of pedestrian traffic injuries treated at a trauma center in Riyadh, highlighting significant demographic, temporal, and clinical patterns. Understanding these trends is essential for optimizing resource allocation and improving emergency care outcomes. Furthermore, the identified age-specific risk factors and seasonal variations underscore the critical need for targeted interventions and policy enhancements to improve road safety and reduce the burden of pedestrian injuries.

## 1. Introduction

Road traffic injuries persist as a major global health issue, causing significant morbidity and mortality. The World Health Organization (WHO) reports that nearly 1.35 million people die annually from traffic-related injuries, with pedestrians comprising a substantial portion of these fatalities [1]. The impact extends to long-term disabilities, high healthcare costs, and significant emotional distress for affected families and communities [2]. Further, pedestrian injuries often require intensive medical intervention and longer hospital stays, imposing further strain on the healthcare system [3]. This challenge is especially severe in rapidly urbanizing nations, where growing vehicle usage significantly increases the risk of pedestrian injuries [4]. Studies from various regions have identified age, time of day, and location as consistent factors influencing pedestrian safety [5,6,7]. Vulnerable groups, particularly the elderly and children, are at greater risk of serious injuries, predominantly occurring during evening hours in urban settings [8].

Several factors contribute to the risk of pedestrian road traffic injuries. Ignoring traffic signals and engaging in jaywalking significantly increase the risk of pedestrian injuries [9,10]. Studies underscore that busy intersections and areas near markets or schools are significant hubs for pedestrian injuries, especially when lacking designated pathways, traffic-calming measures, and proper lighting, leading to an increase in injury rates [11].

Saudi Arabia, with a population of approximately 35.8 million, having one of the highest per capita rates of road traffic injuries globally [12,13], particularly identifies pedestrians as vulnerable [14]. Research highlights numerous safety issues, such as high-speed driving and non-adherence to traffic regulations, exacerbated by rapid urban expansion [14]. Research indicates that young males are particularly vulnerable to pedestrian injuries, aligning with global trends but also presenting unique challenges locally [15,16]. Recent research has concentrated on micro-level factors, such as the effectiveness of crosswalks and public safety campaigns to improve pedestrian safety [17]. Nonetheless, there exists a significant gap in the literature concerning pedestrian injuries in Saudi Arabia.

This research addresses a critical public health issue in Saudi Arabia, aiming to provide a foundation for future preventive strategies and healthcare policies. The primary objectives are to identify patterns in injury timing and their correlation with hospital admissions and outcomes, while pinpointing key risk factors associated with ICU admissions and in-hospital mortality. Additionally, the study seeks to analyze age-specific differences in injury characteristics, seasonal patterns, and outcomes of pedestrian injuries treated at a major trauma center, with a particular focus on demographic trends and clinical outcomes.

## 2. Materials and Methods

The Saudi Trauma Registry (STAR) at King Saud Medical City (KSMC) provided retrospective data for this study. KSMC, a 1400-bed hospital located centrally in Riyadh, annually attends to over 2000 trauma patients in its emergency department (ED) [18]. The data collection process was conducted through the STAR registry, which records information on injury patients who meet specific inclusion criteria. These criteria are as follows: (1) a principal diagnosis of injury, (2) death in the emergency department (ED) following injury, (3) inpatient admission lasting three or more calendar days, (4) inpatient death following injury, or (5) admission to the intensive care unit (ICU). All pedestrian-injured patients who met the registry criteria and were either treated in the KSMC ED or hospitalized due to traumatic injury from August 2017 to December 2022 were included in this study’s analysis. The STAR was utilized to collect data on various aspects, including demographics, injury location and timing, severity and cause of injury, prehospital and hospital-related factors, and in hospital outcomes.

Statistical analyses for this research were conducted using SPSS version 27. In the initial step, primary analysis entailed descriptive analysis. Categorical demographic variables, such as age group, gender, and time of injury, underwent frequency and percentage analysis. Variables such as hospital mode of arrival, disposition from the ED, and injury type were initially converted into categorical variables. Subsequently, frequency analysis in SPSS calculated frequencies and percentages for each group. Continuous variables, such as the Glasgow Coma Scale (GCS), Injury Severity Score (ISS), length of hospital stay (expressed as mean and standard deviation (SD) or median and interquartile range, where appropriate), prehospital physiological assessment (including first systolic blood pressure (BP), first heart rate (HR), and first respiratory rate (RR)), physiological assessment upon arrival at the ED (first systolic BP, first HR, first RR, and first O2 saturation), and length of ICU stay, were described. ICU admission was then transformed into a binary variable (no = <1 day; yes = ≥1 day).

In the second step of the inferential analysis, cross-tabulation analysis was conducted to compare age-specific differences in hospital and ICU admissions and mortality using the chi-square test. Additionally, the same test was applied to examine significant differences in age groups concerning other categorical and continuous variables. To assess gender, age group, mode of arrival, injury type, length of hospital stay, length of ICU stay, ISS, GCS scores, and the necessity of operation upon admission as predictors of ICU admission and mortality, we initially analyzed them using a univariate linear regression model. Following the identification of the predictive value of each variable for ICU admission and mortality, all significant variables were analyzed using a multivariable linear regression model with 95% confidence intervals (CIs). The models were adjusted for covariates, including age, gender, ISS, and GCS scores. A significance value of <0.05 was calculated in both cross-tabulation and regression models. This study was approved by the Institutional Review Board Committee at King Saud Medical City, Riyadh, Saudi Arabia (IRB Reference Number: H1RI-12-June23-01).

## 3. Results

During the study period, a total of 1062 pedestrian-injured patients were admitted to the major trauma center between August 2017 and December 2022. The mean age was 33 ± 17.9 years, with the majority of patients being male (n = 950, 89.5%), aged between 18 and 29 (n = 322, 30.3%) and 30 to 44 (n = 300, 28.2%). Most of the injuries occurred on weekdays (n = 764, 71.9%), predominantly at night between 19:00 and 5:59 h (n = 672, 63.3%), and during the months of April to June (n = 323, 30.4%). Lower extremity injuries were the most commonly reported (n = 498, 27.5%), followed by head injuries (n = 388, 21.3%). More than half of the patients arrived at the hospital by Red Crescent ambulance (n = 533, 53.9%), followed by government ambulance (n = 299, 30.2%). Approximately one-quarter of patients required respiratory assistance (n = 239, 23.9%).

**Figure 1:**
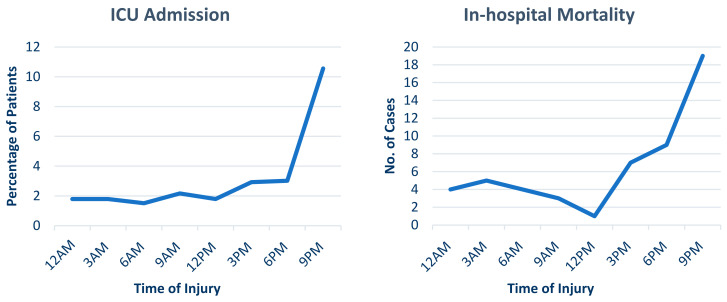
ICU admissions and in-hospital mortality rates by the hour of the injury event.

**Figure 2:**
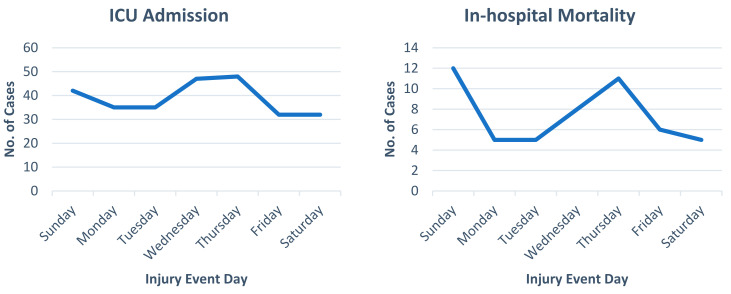
ICU admissions and in-hospital mortality rates by day of the week.

**Figure 3:**
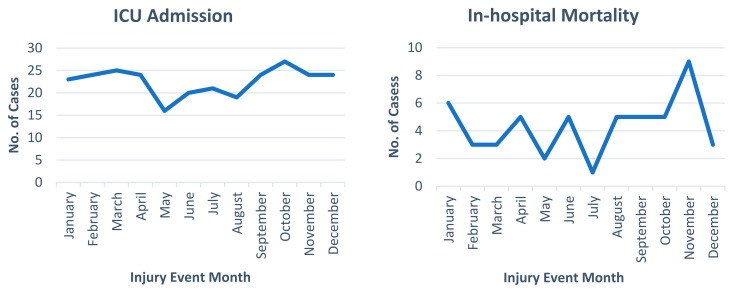
ICU admissions and in-hospital mortality rates by month of the year.

Table 1 displays the demographic, presentation characteristics, and outcomes of pedestrian trauma patients. The median injury severity score (ISS) was nine. Patients with an ISS of 15–40 and >40 numbered n = 301 (28.3%) and n = 19 (1.8%), respectively. The low level of consciousness, indicated by a GCS score of 3–8 and 9–12, was n = 108 (11%) and n = 39 (3.9%), respectively. Regarding disposition from the ED, more than one-third of patients were admitted to the ward (n = 786, 74%), and patients who required operation on arrival were reported in 74 (7%) cases. Over the 5 years, 271 (25.6%) patients were admitted to the ICU, with an overall in-hospital mortality rate of 4.9% (n = 52). Figure 1, Figure 2 and Figure 3 show the frequency of ICU admissions and mortality rates, categorized by the hour, day, and month of injury events, respectively.

The analysis of different age groups revealed that patients aged < 18 were more likely to be involved in weekday crashes compared to weekends (n = 136, 12.8% vs. n = 41, 3.9%, *p* < 0.05). Similarly, crashes among those aged 18–29 were more frequent on weekdays than weekends (n = 231, 21.8% vs. n = 91, 8.6%, *p* < 0.05). Regarding injury type, the majority of lower extremity injuries were observed in the 18–29 and 30–44 age groups (n = 163, 15.3% and n = 141, 13.3%, *p* < 0.26), followed by head injuries (n = 102, 9.6% and n = 100, 9.4%, *p* < 0.5), face and neck injuries (n = 66, 6.2% and n = 43, 4.0%, *p* < 0.5), and then spine injuries (n = 66, 6.2% and n = 78, 7.3%, *p* < 0.05), respectively.

Compared with age-specific differences in the level of consciousness, injury severity, and patient outcome following the injury events, the most observed age group with a level of consciousness (GCS of 3–8) was those aged < 18 (n = 36, 3.7%), followed by patients aged 18–29 years (n = 30, 3.1%), *p* < 0.05. Age groups 18–29 and 30–44 were more likely to present with severe injuries, with ISS between 15 and 40 (n = 76, 7.2% and n = 79, 7.4%, respectively, *p* < 0.05). ICU admission was mostly needed for patients aged 18–29 years (n = 77, 7.3%), followed by those aged < 18 (n = 62, 5.8%) and 30–44 years (n = 62, 5.8%), *p* < 0.05. During the study period, in-hospital mortality was most reported in patients aged ≥60 years (n = 14, 1.3%) and 18–29 years (n = 13, 1.2%), *p* < 0.05. Table 2 illustrates age-specific differences in injury characteristics, seasonality, and outcomes, such as hospital stays, ICU admission, and mortality.

The adjusted predictors for ICU admission and in-hospital mortality are presented in Table 3. The results from the multivariable regression model indicated that ages 30–44 were associated with an increased risk of ICU admission (β = −0.04, 95% CI −0.09 to 0.01, *p* < 0.05), while ages 60 and above were linked to a higher risk of in-hospital mortality (β = 0.10, 95% CI 0.03 to 0.12, *p* < 0.01). Female patients were found to have a higher likelihood of ICU admission (β = −0.03, 95% CI −0.11 to 0.00, *p* < 0.05). Additionally, patients with head injuries (β = 0.08, 95% CI 0.03 to 0.11, *p* < 0.01) and thorax injuries (β = 0.05, 95% CI 0.00 to 0.09, *p* < 0.05) were also more likely to require ICU admission. Furthermore, in-hospital mortality was significantly associated with arrival by private or police vehicle (β = −0.06, 95% CI −0.08 to −0.00, *p* < 0.05), low GCS scores (β = −0.12, 95% CI −0.18 to −0.06, *p* < 0.01), high ISS (β = 0.12, 95% CI 0.00 to 0.01, *p* < 0.001), ICU stays (β = 0.34, 95% CI 0.00 to 0.01, *p* < 0.01), and hospital stays (β = −0.20, 95% CI −0.25 to −0.15, *p* < 0.01).

## 4. Discussion

This study provided a comprehensive analysis of age-specific differences in injury characteristics, seasonal patterns, and outcomes of pedestrian injuries treated at a major trauma center in Riyadh, Saudi Arabia, with a focus on demographic trends and clinical outcomes. The results indicated a significant overrepresentation of young males among the injured, aligning with both international [19] and Saudi-specific studies [20]. This demographic’s predisposition toward risky behaviors, such as jaywalking and using phones while crossing streets, contributes substantially to their elevated injury rates [9,10]. Another potential factor is the higher vehicle speeds, lighting conditions, and the road class, such as outside of the downtown area [21]. These high-speed zones are associated with more severe pedestrian injuries, which could help explain the increased risks faced by young males [22].

The study also revealed that female patients were more likely to require ICU admission, a finding that contrasts with the usual trend of young males being at higher risk for pedestrian injuries. While earlier research typically centered on risk profiles dominated by males [23,24], the results indicated that injuries to females, though less frequent, tend to be more severe, necessitating intensive care. This disparity could be attributed to anatomical differences or societal factors influencing how women engage with traffic environments. These findings provide valuable insights for healthcare providers and policymakers focused on reducing pedestrian injuries in urban settings, emphasizing the need for targeted interventions that address these risky behaviors.

Patients aged 60 and older were observed to have a higher risk of in-hospital mortality after sustaining pedestrian injuries. This observation aligns with broader research showing that older adults are more susceptible to severe outcomes from traumatic injuries. Factors such as reduced bone density, lower physiological reserves, and the presence of comorbidities that hinder recovery could explain this increased vulnerability. Additionally, the decreased mobility typical of this age group might impede their ability to avoid collisions, leading to more severe injuries and an increased risk of in-hospital mortality. Furthermore, older individuals often experience declines in sensory capabilities, such as vision and hearing, which are essential for navigating traffic safely [25].

Regarding clinical characteristics, the study highlighted the severity of pedestrian injuries, which is consistent with previous research both worldwide and specifically in Saudi Arabia [3,26]. The findings showed that a considerable proportion of pedestrian injuries necessitated extensive medical treatments, such as surgeries and extended hospitalizations. Specifically, at KSMC, 7% of the pedestrians needed urgent surgical procedures, and 18.2% were admitted to the ICU, underscoring the serious nature of their injuries. These findings align with other studies that have also reported high rates of severe pedestrian injuries requiring surgical operations and ICU care upon arrival to hospital [3,27].

The research found that younger individuals, particularly those under 18, were more frequently involved in pedestrian accidents during weekdays compared to weekends. This trend may be linked to the morning and afternoon school commutes, suggesting that heavy traffic and possibly insufficient supervision are significant risk factors. Prior studies have consistently noted the susceptibility of younger demographics to pedestrian injuries [28]; however, the specific increase in weekday incidents suggests a complex interaction between daily routines, parental oversight, and community behavior. In Saudi Arabia, where the school week spans from Sunday to Thursday, with the weekend on Friday and Saturday, it is likely that the higher incidence of pedestrian injuries among the 1–18 age group during weekdays relates to their increased presence outdoors during these days, particularly during peak traffic times. This heightened exposure could explain why injuries are more common on weekdays than weekends.

The findings also showed that individuals aged 18–29 were more frequently involved in pedestrian crashes during weekdays than weekends. This age group, typically engaged in activities such as work, college, or other obligations from Monday to Friday, is naturally at greater risk during these days. The daily commitments of these young adults likely increase their exposure to road traffic, which in turn raises their risk of pedestrian–vehicle collisions on weekdays. Additional factors, such as work stress and the urgency to fulfill other responsibilities, could lead to risky behaviors or a reduced focus on road safety [29]. This points to the potential effectiveness of public awareness campaigns targeted at this demographic, emphasizing safe crossing practices and the importance of vigilance while navigating traffic. These age-specific insights highlight the need for preventive measures and public health initiatives to be closely aligned with the daily routines of different age groups. Such tailored interventions could more effectively reduce the risks associated with pedestrian injuries. The influence of the day of the week extends beyond just a time marker—it reflects varying behavioral and environmental factors that heighten the risk of pedestrian injuries among particular age groups.

Adding to the complexity of the results, the study noted that a considerable proportion of pedestrian incidents, nearly one-third (30.4%), occurred in April and June. These months not only conclude the academic year in Saudi Arabia, characterized by heightened school activities, such as exams, but also mark the onset of summer break. Typically, this period could see reduced pedestrian injuries due to fewer school commutes; however, the data show an increase, likely because children and adolescents spend more time outdoors, potentially with less parental supervision, elevating their exposure to traffic-related risks. Additionally, these months witness a rise in domestic and international tourism, possibly introducing tourists unfamiliar with local traffic regulations. While urban areas like Riyadh might see less impact, rural regions could experience more pedestrian traffic due to activities like gatherings. Overlapping with Ramadan and Eid, these months also see heightened nighttime activities [30], potentially increasing pedestrian presence during nighttime. This confluence of factors likely contributes to the observed spike in pedestrian injuries.

This study’s findings carry significant implications for improving outcomes and preventing road crashes, particularly through targeted interventions aimed at vulnerable populations. Identifying high-risk groups, such as young males and school-age children, emphasizes the need for tailored public awareness campaigns and community-based programs. These initiatives could include promoting safe crossing practices, enhancing supervision during school commutes, and enforcing stricter traffic control measures in high-speed zones. Additionally, the study highlighted the importance of gender-sensitive approaches, as evidenced by the disparities in injury severity between males and females. By addressing these specific risk factors, policymakers and healthcare providers can develop focused strategies to reduce the burden of pedestrian injuries in urban areas effectively.

While the study provided valuable insights into pedestrian traffic injuries at a major trauma center in Saudi Arabia, it has several limitations. First, the data were sourced from a single trauma center, which may not be representative of the broader population or other regions within Saudi Arabia. This limits the generalizability of the findings. Second, the retrospective nature of the study relies on the accuracy and completeness of registry data, which may have inherent biases or missing information. Third, the study did not have access to detailed information on the circumstances of each accident, such as the behavior of pedestrians and drivers, environmental conditions, or specific road characteristics, which could provide a deeper understanding of the factors contributing to these injuries. Fourth, the comparison between weekdays and weekends may be influenced by the unequal number of days, which probabilistically leads to more cases on weekdays. Fifth, a portion of the data were collected during the COVID-19 pandemic period, which may have influenced the results due to changes in traffic patterns, healthcare accessibility, and social behaviors during this time. These factors remain limitations of the study and should be considered when interpreting the findings.

## 5. Conclusions

This study underscored the significant burden of pedestrian traffic injuries in Riyadh, highlighting age-specific differences in injury patterns, seasonal trends, and clinical outcomes. Children and young adults were identified as particularly vulnerable groups, especially during weekdays, likely due to school- and work-related activities. The observed seasonal spike in injuries during April and June suggests that factors such as increased outdoor activities, holidays, and tourism may contribute to a higher risk. These findings emphasize the need for targeted public health interventions and safety measures tailored to specific age groups and seasonal variations. Future research should expand to include multiple trauma centers and collect more detailed contextual data to better inform preventive strategies and healthcare policies addressing pedestrian injuries.

## Figures and Tables

**Table 1:** Demographics, presentation characteristics, and patient outcomes following pedestrian injury.

Characteristics	Total, n (%)
Age group	
<17	177 (16.7)
18–29	322 (30.3)
30–44	300 (28.2)
45–59	170 (16.0)
≥60	93 (8.8)
Gender	
Male	950 (89.5)
Female	112 (10.5)
Injury type	
Head and face	388 (21.3)
Thorax	207 (11.3)
Abdomen and pelvis	289 (16.0)
Spine	246 (13.5)
Upper extremities	188 (10.4)
Lower extremities	498 (27.5)
Prehospital physiological assessment, mean (SD)	
First systolic BP	125.71 (21.77)
First heart rate	91.46 (17.40)
First RR	17.45 (3.17)
On arrival at the ED, mean (SD)	
First systolic BP	125 (23.33)
First heart rate	94 (20.910)
First RR	20.17 (2.90)
First O2 saturation	97.02 (5.67)
Assisted respiration	239 (23.9)
GCS score	
13–15	834 (85.1)
9–12	39 (3.9)
3–8	108 (11)
ISS	
≤14	741 (69.8)
15–40	301 (28.3)
>40	19 (1.8)
Requires operation	696 (65.5)
ICU admission	271 (25.6)
Days in hospital, median (IQR)	9 (12)
In-hospital mortality	52 (4.9)

Interquartile range = IQR; Glasgow Coma Scale = GCS; Injury Severity Score = ISS; emergency department = ED; intensive care unit = ICU.

**Table 2:** Age-specific differences in injury characteristics, seasonality, and outcomes.

Variables, n (%)	<18	18–29	30–44	45–59	≥60	*p*-Value
Gender, n (%)						0.036
Male	147 (13.8)	296 (27.9)	272 (25.6)	152 (14.3)	83 (7.8)	
Female	30 (2.8)	26 (2.4)	28 (2.6)	18 (1.7)	10 (0.9)	
Time of day, n (%)						0.166
Day (6:00–18:59)	59 (5.6)	108 (10.2)	112 (10.5)	75 (7.1)	36 (3.4)	
Night (19:00–5:59)	118 (11.1)	214(20.4)	188 (17.7)	95 (8.9)	57 (5.4)	
Day of week, n (%)						0.030
Weekday	136 (12.8)	231 (21.8)	205 (19.3)	133 (12.5)	59 (5.6)	
Weekend	41 (3.9)	91 (8.6)	95 (8.9)	37 (3.5)	34 (3.2)	
Month of injury, n (%)						0.994
January–March	52 (4.9)	82 (7.7)	78 (7.3)	49 (4.6)	26 (2.4)	
April–June	55 (5.2)	95 (8.9)	96 (9.0)	48 (4.5)	29 (2.7)	
July–September	25 (2.4)	53 (5.0)	41 (3.9)	25 (2.4)	14 (1.3)	
October–December	45 (4.2)	92 (8.7)	85 (8.0)	48 (4.5)	24 (2.3)	
Type of injury, n (%)						
Head and face	75 (7.1)	102 (9.6)	100 (9.4)	66 (6.2)	45 (4.2)	0.010
Thorax	33 (3.1)	66 (6.2)	43 (4.0)	40 (3.8)	25 (2.4)	0.049
Abdomen and pelvis	55 (5.2)	72 (6.8)	84 (7.9)	47 (4.4)	31 (2.9)	0.134
Spine	25 (2.4)	66 (6.2)	78 (7.3)	47 (4.4)	30 (2.8)	0.002
Upper extremities	30 (2.8)	56 (5.3)	55 (5.2)	34 (3.2)	13 (6.9)	0.796
Lower extremities	71 (6.7)	163 (15.3)	141 (13.3)	81 (7.6)	42 (4.0)	0.266
GCS						<0.001
13–15	124 (12.6)	255 (26.0)	253 (25.8)	137 (14.0)	65 (6.6)	
9–12	7 (0.7)	10 (1.0)	8 (0.8)	6 (0.6)	8 (0.8)	
3–8	36 (3.7)	30 (3.1)	20 (2.0)	11 (1.1)	11 (1.1)	
Require operation	104 (9.8)	220 (20.7)	200 (18.9)	114 (10.7)	58 (5.5)	0.228
Mean hospital stays	14.12	14.20	14.93	16.94	18.69	0.256
ICU admission (%)	62 (5.8)	77 (7.3)	62 (5.8)	39 (3.7)	31 (2.9)	0.003
Mean ICU stay	5.02	2.54	2.79	3.11	5.54	0.002
Mortality, n (%)	8 (0.8)	13 (1.2)	7 (0.7)	10 (0.9)	14 (1.3)	<0.001

**Table 3:** Adjusted predictors for ICU admission and in-hospital mortality.

Independent Variable	ICU Admission	Mortality
	β (95% CI) *p*-Value	β (95% CI) *p*-Value
Age group		
1–17	−0.00 (−0.05, 0.05) 0.931	−0.05 (−0.06, 0.00) 0.120
30–44	−0.04 (−0.09, 0.01) 0.068	−0.03 (−0.04, 0.01) 0.278
45–59	−0.03 (−0.09, −0.01) 0.129	−0.02 (−0.02, 0.05) 0.421
≥60	−0.03 (−0.11, 0.02) 0.196	0.10 (0.03, 0.12) < 0.001
Gender		
Male	Ref	Ref
Female	−0.03 (−0.11, 0.00) 0.072	−0.01 (−0.56, 0.57) 0.574
Injury type, n (%)		
Head	0.08 (0.03, 0.11) < 0.001	0.01 (−0.03, 0.02) 0.716
Thorax	0.05 (0.00, 0.09) 0.039	0.01 (−0.02, 0.03) 0.733
Spine	−0.00 (−0.05, 0.03) 0.679	−0.01 (−0.02, −0.02) 0.534
Mode of arrival		
Red Crescent ambulance	−0.06 (−0.09, −0.01) 0.010	−0.03 (−0.04, 0.01) 0.221
Private/police vehicle	−0.07 (−0.15, −0.03) 0.003	−0.06 (−0.08, −0.00) 0.048
GCS score	−0.12 (−0.01, −0.00) < 0.001	−0.12 (−0.00, −0.00) < 0.001
ISS	0.17 (0.06, 0.01) < 0.001	0.12 (0.00, 0.01) < 0.001
Length of stay in ICU	0.55 (0.02, 0.03) < 0.001	0.34 (0.00, 0.01) < 0.001
Length of stay in hospital	−0.06 (−0.00, 0.00) 0.022	−0.20 (−0.00, −0.00) < 0.001
Require operation	−0.00 (−0.4, 0.03) 0.926	−0.04 (−0.04, 0.00) 0.118

## Data Availability

The datasets used during the current study are available from the corresponding author upon reasonable request.
